# Non-uniform evolutionary response of gecko eye size to changes in diel activity patterns

**DOI:** 10.1098/rsbl.2018.0064

**Published:** 2018-05-23

**Authors:** Lars Schmitz, Timothy E. Higham

**Affiliations:** 1 W. M. Keck Science Department, Claremont McKenna, Scripps, and Pitzer Colleges, Claremont, CA 91711, USA; 2 Dinosaur Institute, Natural History Museum of Los Angeles County, Los Angeles, CA 90007, USA; 3 Department of Evolution, Ecology, and Organismal Biology, University of California Riverside, Riverside, CA 92521, USA

**Keywords:** geckos, eye size, vision, adaptive evolution, diel activity pattern, habitat clutter

## Abstract

Geckos feature a large range of eye sizes, but what drives this phenotypic diversity is currently unknown. Earlier studies point towards diel activity patterns (DAPs) and locomotory mode, but phylogenetic comparative studies in support of the proposed adaptive mode of eye evolution are lacking. Here, we test the hypothesis of DAPs as the driver of eye size evolution with a dataset on 99 species of gecko. Results from phylogenetic generalized least-square analysis (PGLS) and multivariate model-fitting reveal smaller eyes in diurnal geckos consistent with different phenotypic optima. However, Bayesian analyses of selective regime shifts demonstrate that only two of nine transitions from nocturnal to diurnal activity are coupled with decreases in eye size, and two other regime shifts are not associated with DAP transitions. This non-uniform evolutionary response suggests that eye size is not the only functionally relevant variable. Evolutionary adaptations may therefore include different combinations of several traits (e.g. photoreceptors), all with the same functional outcome. Our results further demonstrate that DAP only partially explains eye size diversity in geckos. As open habitats favour the evolution of large eyes while obstructed habitats favour small eyes, the degree of habitat clutter emerges as another potential axis of eye diversification.

## 1. Introduction

One of the grand challenges in biology is to elucidate the origins of phenotypic diversity [1–4]. Integrative functional and evolutionary analyses can solve this challenge by connecting organismal shape and function with specific niche dimensions, with promise for identifying the mechanisms that drive morphological evolution [3]. Geckos are ideal for pursuing the quest for the origins of phenotypic diversity, as they are known to harbour many lineages, forms and functions, occupying habitats that build the centre stage for an unparalleled evolution of biodiversity within squamates [5].

Geckos feature a large range of eye sizes, both in absolute and relative terms [6,7]. Eye size affects both visual acuity and visual light sensitivity [8], but larger eyes come with higher metabolic cost [9]. Large eyes also reduce available space for jaw adductor muscles and may decrease skull stability [10]. Therefore, gecko eye size evolution is expected to be governed by an evolutionary trade-off between selective benefits of visual performance and the cost associated with larger eyes. This evolutionary trade-off unfolds in the context of photic environments that are largely controlled by diel activity pattern (DAP).

The origin of geckos is nocturnal, but there are many independent evolutionary transitions to diurnal and cathemeral/crepuscular activity patterns [11], offering repeated opportunities to observe organism–environment coevolution. Given the disparate light levels during night and day, the phenotypic diversity of the gecko visual system is expected to be substantially influenced by DAP. While published data substantiate an association between nocturnal activity and large eyes [6,7], it is unknown if these eye size differences arise within evolutionary transitions between DAPs, as expected in an adaptive scenario. In addition, ground-dwelling geckos can also have large eyes [6,7], pointing towards a more complex adaptive landscape of eye size evolution in geckos than suggested by the nocturnal–diurnal axis.

We tested the hypothesis that DAPs are the main drivers of eye size evolution in geckos, and predicted that evolutionary transitions to diurnality are coupled with reduction of eye size, reflecting the evolutionary trade-off between visual performance and the metabolic and biomechanical cost. Surprisingly, our phylogenetic perspective reveals that the morphological response to independent evolutionary DAP transitions is not uniform.

## 2. Material and methods summary

We used data on anteroposterior eye diameter (ED) and snout-vent-length (SVL) for 99 gecko species, representing a mix of the literature data (*n* = 37) and new data collections on museum specimens (*n* = 62; 1–12 individuals/species), and compiled information on DAP (electronic supplementary material). Comparative analyses were performed on species averages in a phylogenetic context [12] using R v. 3.4.4 [13]. We reconstructed the evolutionary history of DAP with stochastic character mapping in phytools [14]. Eye size disparity between species with different DAPs was assessed by phylogenetic generalized least-square analysis (PGLS) [15], and multivariate model-fitting [16] provided insights regarding whether a model with independent adaptive peaks for each DAP fitted the data better than a single-peak model. To investigate the adaptive landscape of residual eye size (calculated from PGLS) in more detail, we adopted the Bayesian implementation of the Ornstein–Uhlenbeck model of trait evolution (bayou, [17]), an agnostic approach to identify selective regime shifts over a phylogeny (see the electronic supplementary material).

## 3. Results and discussion

**Figure 1.**
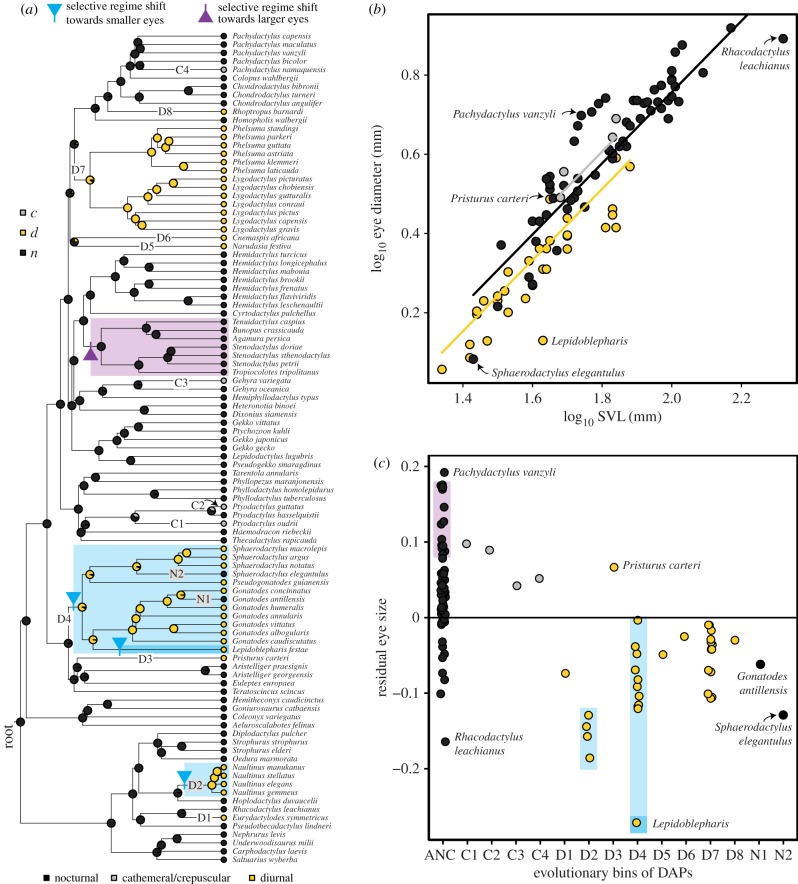
(*a*) Evolution of DAP in geckos, summarized from 1000 iterations of stochastic character mapping, and bayou-identified selective regime shifts for size-corrected ED. Blue identifies clades with a shift towards selection of smaller residual eye size, while purple identifies shifts towards larger residual eye size. D1–8 refer to individual evolutionary transitions to diurnal activity, C1–4 are transitions to cathemeral/crepuscular activity and N1–2 identify shifts from diurnal to nocturnal. (*b*) Plot of log_10_-transformed ED against log_10_-transformed SVL, with fitted lines from a PGLS model with DAP as treatment. The different intercepts are consistent with different phenotypic optima. Diurnal species tend to have small eyes, a pattern also visible in (*c*), where residual eye size is ordered according to evolutionary bins of DAP. ANC, ancestrally nocturnal geckos.

Stochastic character mapping of DAPs confirms a nocturnal origin of geckos with several independent transitions to diurnal and cathemeral/crepuscular activity (figure 1*a*; [12]). Our dataset of 99 gecko species contains evidence for nine independent transitions to diurnality (D1–8), four transitions to cathemeral/crepuscular behaviour (C1–4) and two transitions from diurnal to nocturnal activity (N1–2). Two of the diurnal transitions represent deep radiations: *Sphaerodactylus*/*Gonatodes* (D4, 95.7 Myr) and *Phelsuma*/*Lygodactylus* (D7, 90.3 Myr). The *Naultinus* clade represents a younger transition (D2, 6.3 Myr). All other transitions are represented by single lineages.

Diurnal geckos have smaller eyes for given SVL than other geckos. PGLS results support that the eyes of geckos with different DAPs scale with negative allometry (0.87; similar to other vertebrates [18]), but have different intercepts. The PGLS model with DAP as treatment (ED ∼ SVL + DAP) received stronger support than the model without treatment (ED ∼ SVL, ΔAICc = 15.9). Multivariate model-fitting suggests that these different intercepts are consistent with different phenotypic optima (mean ΔAICc = 23.2, averaged over 1000 stochastic character maps). However, there is overlap in eye size between DAP groups, congruent with findings in other vertebrates [19–21].

We explored this overlap by an approach dictated by independent evolutionary transitions between DAPs. First, we calculated the residuals of all species from a simple PGLS model (eye size ∼ SVL) and then binned residuals according to DAP transitions (figure 1*c*). All diurnal bins contain smaller residual eye size than expected, except for *Pristurus carteri* (D3). All four cathemeral/crepuscular lineages feature relatively large eyes, whereas the tertiary nocturnal lineages have small residual eye size, similar to their diurnal relatives, D4. Ancestrally nocturnal geckos (ANC) have large residual eye sizes, but there is overlap with diurnal bins. The overlap suggests that DAP is not the only driver of eye size evolution, and/or gecko eyes follow different evolutionary trajectories in adapting to different light levels.

The pattern of different evolutionary trajectories is reinforced by the analysis of the adaptive landscape. Four strongly supported selective regime shifts emerged, and while two of these shifts are congruent with our prediction, the other two are surprising. Geckos enter regimes selecting smaller eyes at D2 (*Naultinus*) and D4 (*Sphaerodactylus*/*Gonatodes*), but none of the other transitions to diurnality are characterized by a change in selection for smaller eyes. However, an additional shift towards smaller eyes falls on the branch leading to *Lepidoblepharis*, the taxon with the relatively smallest eyes in the data, nested within the *Sphaerodactylus*/*Gonatodes* clade. The fourth strongly supported regime shift is most surprising, situated within a radiation of nocturnal geckos, at the branch leading to the Palearctic naked-toe geckos. These geckos enter a regime that favours the evolution of even larger eyes than those of their immediate relatives (figure 1*b,c*). Our results, therefore, reveal a non-uniform evolutionary response in iterated DAP transitions and also suggest that DAP alone is insufficient to fully explain eye size evolution in geckos.

What other factors determine the adaptive landscape of gecko eye size evolution? Locomotory mode may influence eye size, with ground-dwelling species having larger eyes than climbing species [6,7]. However, the geckos with some of the largest (Palearctic naked-toe geckos) and smallest (*Lepidoblepharis*) eye size in our data are ground-dwelling, and the mechanistic link between locomotor mode and visual performance is not well documented [6], especially given that many geckos both run and climb [22].

We propose that habitat clutter is an additional important environmental factor that impacts eye evolution. Palearctic naked-toe geckos occupy (semi-)arid habitats with little vegetation cover, providing an unobstructed, long-range view, whereas *Lepidoblepharis* is active in leaf-litter on the ground [22], with obstructed vision and short viewing distances. Optical modelling has shown that large eyes enable larger visual ranges in both diurnal and nocturnal settings [23], hence larger eyes can provide a selective benefit by extending the target detection distance when emerging from a cluttered to an open habitat. Accordingly, Palearctic naked-toe geckos, released from the constraints of obstructed habitats, may have evolved larger eyes with longer visual ranges. The reverse evolutionary transition, from uncluttered to obstructed habitats, likely results in selection against large eyes, which we may see in *Lepidoblepharis*, where leaf-litter constrains visual range. Precise habitat clutter data for geckos are unavailable, hence a formal test of this hypothesis is still wanted, but it is possible that the ground-dwelling/climbing axis of eye diversity [6] is related to habitat clutter, as ground-dwelling specialists tend to be more common in open habitats [22]. We propose that habitat and DAP jointly drive visual range, locomotor speed [24,25], and, ultimately, eye size evolution, offering a new perspective on Leuckart's Law [26]. However, even with full understanding of the photic environment, eye size can follow different evolutionary trajectories, as size is not the only determinant of visual performance. Eyes represent a many-to-one mapping system of structure to function [3], and future studies should integrate across different traits, including optics and retina structures.

## Supplementary Material

Detailed information on data collection and methods

## Data accessibility

All data and code are available on the Dryad Digital Repository: http://dx.doi.org/10.5061/dryad.96qr7n2 [27].
