# Supplementary material for: Non-uniform evolutionary response of gecko eye size to changes in diel activity patterns
Source: Biol Lett. 2018 May 23;14(5):20180064. doi: 10.1098/rsbl.2018.0064 (PMC6012706; doi:10.1098/rsbl.2018.0064)
Supplement: Detailed information on data collection and methods [file rsbl20180064supp1.pdf]

Non-uniform evolutionary response of gecko eye size to changes in diel activity patterns

Lars Schmitz*, W.M. Keck Science Department, Claremont McKenna, Scripps, and Pitzer Colleges, Claremont, CA 91711, USA

Timothy E. Higham, Department of Evolution, Ecology, and Organismal Biology, University of California Riverside, Riverside, CA 92521

*corresponding author, lschmitz@kecksci.claremont.edu

Electronic Supplementary Material

Eye and body size measurements, diel activity pattern

We used a total number of 99 gecko species for this study (table S1, [1]), representing a mix of literature data and new data collections. We measured the anteroposterior eye diameter (ED) and snout-vent-length (SVL) in 62 gecko species (1-12 mostly adult individuals per species, with a mean of 2.5 individuals per species) in museum collections. All specimens were in vitro, i.e., animals were preserved in formaldehyde and subsequently stored in 70% ethanol). ED is considered to be tightly correlated with the true diameter of the eye, as the eye is well discernable even in geckos with eyelids. We supplemented these data with published records for 37 species [2]. SVL of all species in the sample ranged from 21.92 to 208.5 mm.

We compiled information on their diel activity pattern (DAP) through many different sources, including the primary literature (e.g., [3]), natural history books [4], the IUCN red list [5], and personal observation (TEH). Using categories as defined by Schmitz and Motani [6], we recognize 61 nocturnal, 4 cathemeral/crepuscular, and 34 diurnal species in our sample.

Phylogenetic comparative analysis

All comparative analyses were performed on species averages in a phylogenetic context [7] using R 3.4.4 [8]. This time-calibrated tree was estimated with maximum likelihood (RAxML)

from data on 52 genes for 4162 squamate species [7], and we pruned this tree to match our data. We lacked alpha taxonomic data for two taxa, *Lepidoblepharis* sp. and *Pseudogonatodes* sp. To include them into the phylogenetic comparative analysis we swapped in representative lineages of these clades (*Lepidoblepharis festae* and *Pseudogonatodes guianensis*). We initially explored the ancestral states of diel activity pattern (3 states: nocturnal, cathemeral/crepuscular, and diurnal) with the ace-function in the ape package [9]. We tried the equal-rates (ER), the symmetric-rates (SYM), and the all-rates-different (ARD) model. Both SYM and ARD models caused computational difficulties, probably because of too many states (3) for the given number of tips (99). We therefore restricted all subsequent ancestral state reconstructions to the ER-model, which suggested a nocturnal origin of geckos, consistent with a recently published study [10]. Next, we reconstructed the evolutionary history of DAP with stochastic character mapping, assuming equal transition rates, summarized from 1000 iterations implemented in phytools [11].

Sample size likely influences ancestral state reconstructions. To test whether our current reconstructions for 99 species were reliable, we combined previously published data [10] with our current dataset and ran a more comprehensive analysis with 254 species (table S2, [1]). As before, we applied stochastic character mapping with equal transition rates (figure S1). Next, we pruned the tree summarized from 1000 SIMMAP iterations and compared the node estimates with our previous version obtained from 99 species. These node estimates were the same. As we need full SIMMAP trees for the mvMORPH analysis (see below), we proceeded with the ancestral state reconstruction for 99 species.

Eye size differences between groups of DAP were assessed by phylogenetic generalized least squares (PGLS, [12, 13]). We determined the extent of phylogenetic autocorrelation in the data by estimating phylogenetic signal with two different approaches. First, we focused on Pagel's λ, a tree transformation parameter that multiplies the off-diagonals of the correlation matrix. For $\lambda=1$, the common descent of species explains the trait pattern well, i.e., phylogenetic signal is strong, while for $\lambda<1$ the amount of phylogenetic signal decreases.

We estimated Pagel's λ for the residual errors in the generalized least square model with maximum likelihood, using the ape function corPagel() in conjunction with nlme's gls() function [9, 12, 13]. Second, we tried α, one of the parameters of the Ornstein-Uhlenbeck (OU) model, to estimate phylogenetic signal. The α value is a parameter that describes the strength of selection, with large α describing strong selection and weak phylogenetic signal, and small α describing weak selection and strong phylogenetic signal. We estimated α for the residual errors in the generalized least square model with maximum likelihood, using the ape function corMartins() in conjunction with nlme's gls() function [9, 12, 13].

For all PGLS regressions with Pagel's (Brownian Motion, BM) correlation structure, we verified that the maximum likelihood estimate of Pagel's λ did represent a global optimum, and checked for normal distribution of residuals and absence of heteroscedasticity. When attempting PGLS with an OU correlation structure, problems with the likelihood calculations emerged, which we tried to solve by selecting different starting values of α (α>1.6). However, the estimated parameter value was never different from the starting value. When performing PGLS over a range of fixed α values (without maximum likelihood optimization), we found a flat log likelihood distribution, with large log likelihood only for very small α values, therefore approaching a simple BM model. Hence, we restricted our full analysis to PGLS with BM correlation structure.

To determine if DAP influences ED, we fitted two basic PGLS models to the data. One in which ED solely depends on SVL (ED ~ SVL), and one in which DAP was added as a treatment (ED ~ SVL + DAP). We selected the better fitting model by calculating sample-size corrected Akaike Information Criterion (AICc) scores. The model with lower score is better, and considered strongly preferred over alternative models if delta AICc is >4 [14]). Our results show that PGLS with DAP as treatment is the better fitting model (ΔAICc=15.9), suggesting the presence of different phenotypic optima for each group of activity pattern.

We tested for the presence of different phenotypic optima with a multivariate model-fitting approach (mvMORPH, [15]) that we performed over the full set of 1000 SIMMAP reconstructions. Specifically, we tested whether a model with multiple peaks (OUM), one for each group of DAP, fits data better than a single peak model (OU1). AICc scores identify the OUM model as the best models, with ΔAICc for the 1000 iterations averaging at 23.2±5.4, ranging from 3.5 to 36.1. Simulations demonstrate that the type I error rate is reasonably low (0.1) and the power is high (1), averaged over 100 iterations. The estimated θ (optimal trait value) for diurnal species are SVL= 27.6±1.1mm with ED=1.6±1.2mm, and SVL=42.5±1.4mm with ED=5.0±1.34mm for cathemeral/crepuscular species. Estimated θ values for SVL (69±1.1mm) and ED (6.3±1.1mm) are largest for nocturnal species.

To investigate the adaptive landscape of residual eye size (calculated as residuals from a PGLS with BM correlation structure) in more detail, we adopted the Bayesian implementation of the Ornstein-Uhlenbeck model of trait evolution (bayou, [16]), an agnostic approach to identify selective regime shifts across all branches of a phylogeny. We multiplied residuals by 10 to avoid computational issues that may emerge from small trait values and long branches. Probabilistic priors for α (a measure for the strength of selection) and σ^2 (the evolutionary rate parameter) were left at defaults, but the prior for θ (the optimal trait value) was drawn from a normal distribution around the trait mean with a standard deviation of 5. We set the expected number of selective regime shifts to 20, with an upper limit of 196, the number of branches in the tree, where each branch had the same probability to feature a shift, irrespective of its length. The reversible jump Markov chain Monte Carlo simulations (two independent chains) were run for 2,000,000 generations, of which the first 30% was discarded as burn-in. We ensured prior adequacy by verifying that the estimated parameters (σ^2 and α) were not exactly congruent with the priors, and checked for convergence with Gelman's R (for log likelihood, σ^2, and α) and a plot of the posterior probabilities for shifts along branches from each chain against each other

(figure S2a, b). Gelman's R compares the variance within and between different MCMC chains by calculating a scale reduction factor, which approaches 1 if convergence is reached [17]. We considered selective regime shifts well-supported if their respective posterior probability was far outside the main distribution of all branches (figure S2c). Similar to the mvMORPH analysis, the estimated θ values were well within the range of the empirical data. What follows are parameter estimates from a single MCMC run, but estimates are similar across different chains. The ancestral θ is estimated at 0.019 (residual eye size, please also compare to Figure 1c). The *Naultinus* clade (D2) has an estimated θ of -0.135, while the *Sphaerodactylus*/*Gonatodes* clade (D4) has a θ of -0.07, except for *Lepidoblepharis* which is characterized by a regime with a θ of -0.34. The *Lepidoblepharis* θ is lower than the observed residual, which may suggest that bayou has difficulties estimating θ for this extremely small residual eye size. The estimated θ for the Palearctic Naked-Toe Geckos (0.158) is well within the observed range. The global estimate of α (strength of selection) is 3.3, with a large standard deviation (±2.7), which means that the phylogenetic half-life (log(2)/α, the time required to evolve halfway from the ancestral state toward a trait optimum) may range from ca. 0.1 to 1.1 million years.

References

1. Schmitz L, Higham T. 2018 Data from: Non-uniform evolutionary response of gecko eye size to changes in diel activity patterns. Dryad Digital Repository. (doi:10.5061/dryad.96qr7n2)

2. Werner YL, Seifan T. 2006 Eye size in geckos: Asymmetry, allometry, sexual dimorphism, and behavioral correlates. Journal of Morphology 267, 1486–1500. (doi:10.1002/jmor.10499)

3. Krause V. 2013 A new species of the genus *Tropiocolotes* Peters, 1880 from western Iran (Squamata: Sauria: Gekkonidae). Zootaxa 3716, 22. (doi:10.11646/zootaxa.3716.1.2)

4. Malhotra A, Thorpe RS. 1999 Reptiles & Amphibians of the Eastern Caribbean. London and Oxford: Macmillan Educ., Ltd.

5. IUCN 2017. IUCN Red List of Threatened Species. Version 2017.3. <www.iucnredlist.org>. Downloaded on 21 January 2018.

6. Schmitz L, Motani R. 2010 Morphological differences between the eyeballs of nocturnal and diurnal amniotes revisited from optical perspectives of visual environments. Vision Research 50, 936–946. (doi:10.1016/j.visres.2010.03.009)

7. Zheng Y, Wiens JJ. 2016 Combining phylogenomic and supermatrix approaches, and a time-calibrated phylogeny for squamate reptiles (lizards and snakes) based on 52 genes and 4162 species. Molecular Phylogenetics and Evolution 94, 537–547. (doi:10.1016/j.ympev.2015.10.009)

8. R Core Team. 2017. R: A language and environment for statistical computing. R Foundation for Statistical Computing, Vienna, Austria. URL https://www.R-project.org/.

9. Paradis E, Claude J, Strimmer K. 2004 APE: Analyses of Phylogenetics and Evolution in R language. Bioinformatics 20, 289–290. (doi:10.1093/bioinformatics/btg412)

10. Gamble T, Greenbaum E, Jackman TR, Bauer AM. 2015 Into the light: diurnality has evolved multiple times in geckos. Biological Journal of the Linnean Society 115, 896–910. (doi:10.1111/bij.12536)

11. Revell LJ. 2011 phytools: an R package for phylogenetic comparative biology (and other things). Methods in Ecology and Evolution 3, 217–223. (doi:10.1111/j.2041-210x.2011.00169.x)

12. Symonds MRE, Blomberg SP. 2014 A Primer on Phylogenetic Generalised Least Squares. In Modern Phylogenetic Comparative Methods and Their Application in Evolutionary Biology, pp. 105–130. Springer Berlin Heidelberg. (doi:10.1007/978-3-662-43550-2_5)

13. Pinheiro J, Bates D, DebRoy S, Sarkar D, and R Core Team. 2017 nlme: linear and nonlinear mixed effects models. R package version 3.1-131, URL https://CRAN.R-project.org/package=nlme.

14. Burnham KP, Anderson DR. 2003 Model selection and multimodel inference, a practical information-theoretic approach. New York, Springer.

15. Clavel J, Escarguel G, Merceron G. 2015 mvmorph: an R package for fitting multivariate evolutionary models to morphometric data. Methods in Ecology and Evolution 6, 1311–1319. (doi:10.1111/2041-210x.12420)

16. Uyeda JC, Harmon LJ. 2014 A novel Bayesian method for inferring and interpreting the dynamics of adaptive landscapes from phylogenetic comparative data. Systematic Biology 63, 902–918. (doi:10.1093/sysbio/syu057)

17. Gelman A, Rubin DB. 1992. Inference from iterative simulation using multiple sequences. Statistical Science 7, 457–511.

Figure captions

Figure S1. Evolution of diel activity patterns in geckos inferred from data on 254 species (table S2). Pie charts at the internal nodes represent node estimate summaries of 1000 stochastic character maps.

Figure S2. a) Gelman's R values as function of reversible jump MCMC generation for log likelihood (top panel), α (center), and σ^2 (bottom). Convergence is reached, i.e., both dashed and solid lines are flat, without jumps, after about 600,000 generations. We discarded all results as burn-in to the left of the red dashed line. b) We plotted the posterior probabilities of a selective regime shift along a given branch obtained from each reversible-jump MCMC chain against each other to test whether the two chains provided similar results. The bivariate plot strongly suggests that the chains had reached stationarity, as the points deviate only very slightly from the expected slope of 1 (solid line). c) Density distribution of all posterior probabilities (mean from the two chains) for selective regime shifts. The expected probability of a selective regime shift (1/(2*n-2), where n is the number of species) is indicated by the dashed line. We considered 4 regime shifts well supported as they were far outside the main distribution, as indicated by the blue vertical arrow (posterior probabilities: 0.97, 0.96, 0.91, and 0.88).

Figure S1.

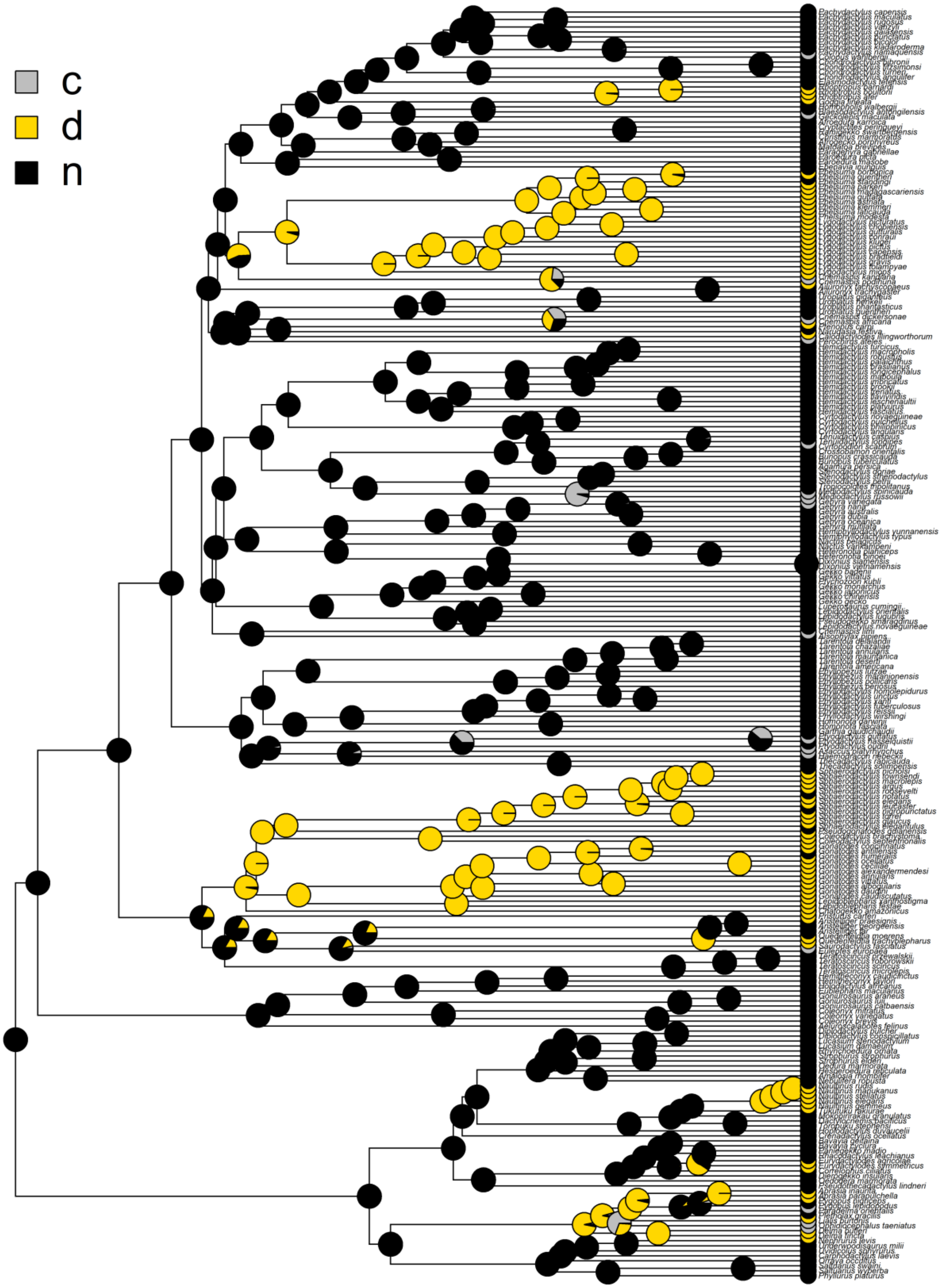

Figure S2.

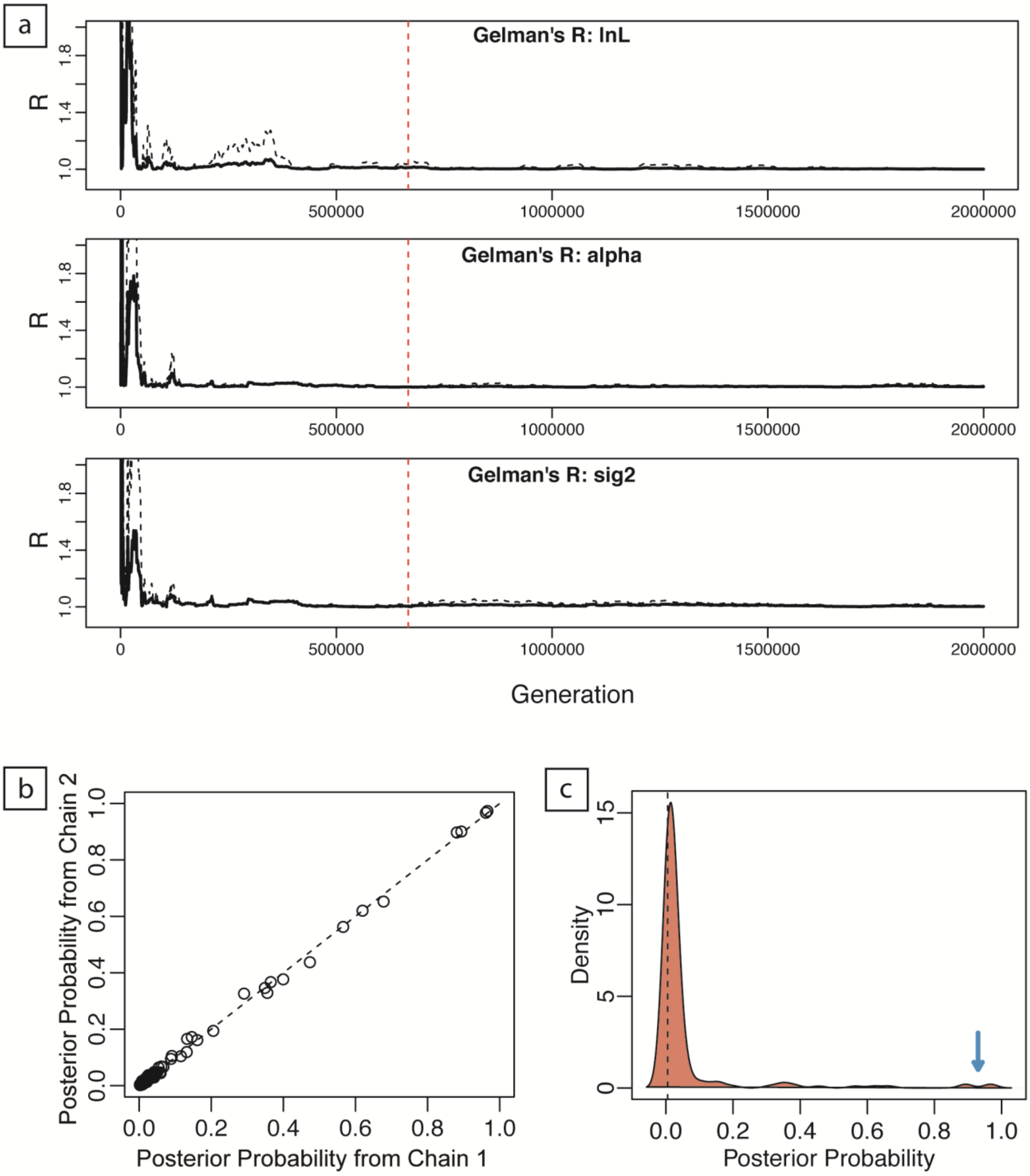
a
Gelman's R: lnL
R
1.0
1.4
1.8
0
500000
1000000
1500000
2000000
Gelman's R: alpha
Gelman's R: sig2
Generation
b
Posterior Probability from Chain 2
Posterior Probability from Chain 1
0.0
0.2
0.4
0.6
0.8
1.0
c
Density
0
5
10
15
Posterior Probability
